# Comparability of (Post-Concussion) Symptoms across Time in Individuals after Traumatic Brain Injury: Results from the CENTER-TBI Study

**DOI:** 10.3390/jcm11144090

**Published:** 2022-07-14

**Authors:** Diego Rivera, Sven Greving, Juan Carlos Arango-Lasprilla, Nicole von Steinbuechel, Marina Zeldovich, CENTER-TBI Participants and Investigators

**Affiliations:** 1 Department of Health Sciences, Public University of Navarre, Arrosadia Campus, 31006 Pamplona, Spain; diegoriveraps@gmail.com; 2 Instituto de Investigación Sanitaria de Navarra (IdiSNA), Irunlarrea Street 3, 31008 Pamplona, Spain; 3 Institute of Medical Psychology and Medical Sociology, University Medical Center Göttingen, Waldweg 37A, 37073 Göttingen, Germany; sven.greving@med.uni-goettingen.de (S.G.); nvsteinbuechel@med.uni-goettingen.de (N.v.S.); 4 Department of Psychology, Virginia Commonwealth University, Richmond, VA 23284-2018, USA; jcalasprilla@gmail.com

**Keywords:** post-concussion symptoms, Rivermead Post-Concussion Symptoms Questionnaire, measurement invariance, longitudinal assessments, traumatic brain injury

## Abstract

Post-concussion symptoms often occur after TBI, persist and cause disabilities. The Rivermead Post-Concussion Symptoms Questionnaire (RPQ) is widely used in this population, but little is known about the comparability of the symptoms over time, i.e., longitudinal measurement invariance (MI). The objectives of this study were to analyze the longitudinal MI of RPQ symptoms from three to twelve months after TBI and to find factors related to RPQ symptoms. The study involved 1023 individuals after TBI who took part in the Collaborative European NeuroTrauma Effectiveness Research in TBI (CENTER-TBI) study and completed the RPQ at three, six and twelve months post-injury. Longitudinal confirmatory factor analysis showed that the three-factor structure (somatic, emotional and cognitive) remains stable within one year after TBI. Linear mixed models revealed that sex, injury cause and prior psychiatric problems were related to the RPQ three-factor structure as well as to the RPQ total score. The study strengthens evidence for the RPQ’s factorial structure stability within one year after TBI and identifies sex, injury cause and prior psychiatric problems as important factors that may help clinicians to prevent future complications of symptomatology after TBI.

## 1. Introduction

Traumatic brain injury (TBI) is one of the main causes of disability and death in young people and adults between the ages of 18 and 35, affecting 69 million people each year worldwide [1]. In Europe, the incidence of TBI varies by country, from 47.3 per 100,000 population per year (Spain) to 694 per 100,000 population per year (Republic of San Marino) [2]. Advancements in emergency and intensive care services in recent decades have increased survival. Evaluation and diagnostic protocols identifying problems shortly after a TBI can facilitate timely intervention to prevent future complications or sequelae.

TBI can cause physical, cognitive and emotional consequences in the short and the long term. Physical problems usually include headaches [3,4,5,6,7,8], nausea [9,10,11,12,13], dizziness [3,4,5,14,15,16], sensitivity to light or noise [9,12,15,17], blurred or double vision [3,5,11,15,18,19,20] and fatigue [4,8,15,16,21,22,23,24,25]. Cognitive alterations include problems in processing speed, attention and concentration [9,13,15,24,26,27,28,29,30]; executive functions [20,28,31,32,33,34]; learning and memory [13,17,20,26,34,35,36,37]; and language [26]. Individuals after TBI usually report symptoms of depression [5,15,19,28,30,38,39,40,41,42,43,44,45,46], suicidal ideation [47,48,49], anxiety [15,30,38,42,43,45,46] and post-traumatic stress [50]. Furthermore, emotional lability and apathy have been noted [5,25,30,51,52]. Regarding the behavioral consequences of TBI, individuals also experience irritability [4,28,38,45,53,54,55], aggressiveness [55], and, in some cases, personality changes [25,56,57], restlessness [3,11,45,58] and insomnia [16,23,28,59,60,61].

These symptoms are referred as to post-concussion symptoms. They often occur after mild to moderate TBI [62]. However, individuals after severe TBI also suffer from comparable deficits [45]. These TBI-related complaints usually resolve within a period of three months [63]. Nevertheless, some deficits may persist for up to one year after injury [64]. If not treated in time, they could last longer than expected and negatively impact other areas of the patient’s life to the point of causing disability [65,66]. For example, individuals after TBI are known to have a poorer quality of life compared to people without TBI [15,67,68,69]. Furthermore, even though most individuals after mild TBI return to work [70], there is evidence that those with more severe injuries have difficulties or worse job performance [71,72]. In addition, some individuals after TBI report difficulties in returning to their daily routine [73,74] and even driving, with less anticipation of accidents compared to healthy people [75,76].

Several factors have been repeatedly found to be associated with short- and long-term prognoses of these symptoms after TBI. Some of the most important sociodemographic characteristics include age [4,5,9,16,20,24,25,29,44,45,77,78,79,80,81,82,83], gender/sex [4,5,9,16,17,19,20,24,25,29,37,44,81,82,84,85,86], living situation [15,24], employment status [5,24,80,82], marital status [5,16,19,82,86], education [9,11,20,24,28,70,82,84], ethnicity/race [9,16,19,28,30,37,80,82,87,88] and socioeconomic class [25,80]. Clinical factors including motor response [78,79,80,89], comorbidity [80,84,90], loss of consciousness (LOC) [4,15,81,91], a number of previous concussions [5,25,81,82,84], amnesia [25,28,29,81], total score on the initial Post-Concussion Scale [81,82], psychiatric history [4,5,11,25,44,70,83], alcohol abuse [16,44,82,92], illicit drug abuse [44,82,93,94], attention deficit hyperactivity disorder [25,37], TBI severity [24,34,77,82,83,95,96], depressive disorder [8,19,25,28], anxiety disorder [25,97], stress disorder [8,13,17] and the mechanism of injury [4,16,29,82,84,90,98] have been shown to be associated with symptom burden after TBI.

Post-concussion symptoms may vary depending on the time after injury and the instruments used to measure predictors and outcomes. Research has typically evaluated individuals after TBI at one time point, for instance, at one month [4,16,25,29,30,82,99], between two and six months [25,84], at six months [11,13,20,30,96] and at one year [5,30,80,99]. Furthermore, longitudinal assessments of symptoms and predictors following TBI often use unsystematic time points within a few weeks after TBI [37]. Studies use different instruments such as the Rivermead Post-Concussion Symptoms Questionnaire (RPQ) [4,15,16,22,24,29,30,39,45,80,99], Neurobehavioral Symptom Inventory (NSI) [19,20,28,82,96], Beck Depression Inventory (BDI II/BDI-III) [19,49,99], Alcohol Use Disorders Identification Test (AUDIT) [44,99] and neuropsychological batteries/tests, such as the WAIS-III (Wechsler Adult Intelligence Scale [26,34,35,36,44], Trail Making Test part A and B (TMT-A, B) [9,26,35,36], Colour-Word Interference Test [26,34,35,36], Conners’ Continuous Performance Test [35,36], Delis Kaplan Executive Function System [34,35,36] and California Verbal Learning Test–II [34,35,36], among others.

Among these instruments, the RPQ is commonly used in patients after TBI, as suggested by the Common Data Elements (CDA) recommendations [100,101] to monitor post-TBI symptoms in research and clinical practice [62]. The RPQ has been originally declared as a unidimensional measure [62] consisting of 16 symptoms rated on a five-point Likert scale (from 0 = “not experienced at all” to 4 = “a severe problem”). However, the questionnaire has been subjected to further analyses indicating the multidimensionality of the construct [102,103,104,105,106]. In a recent cross-sectional study on the comparability of the RPQ across six languages and TBI severity groups using six-month CENTER-TBI data [107], the authors found that the factorial structure of the RPQ structure proposed by Smith-Seemiller et al. [102] outperformed competing factorial solutions (i.e., [62,104,106,108]) with respect to data fit. This solution includes three factors (somatic, emotional and cognitive) that can provide additional information on impairment in individual domains. However, despite the researchers agreeing that the RPQ is a non-unidimensional measure, no consensus has been achieved on which factorial solution should be applied for the scoring.

Unfortunately, there is—to our knowledge—relatively little evidence on how RPQ scores change over predefined times after a TBI. For the clinical administration of the RPQ over time and the follow-up assessment of post-concussion symptoms, it is necessary to provide empirical evidence on whether the questionnaire retains its factorial structure over time. Whenever variables are assessed at different time points, it is assumed that changes in the variables are solely attributable to the changes in time. To verify whether this assumption holds true, it is important to ascertain measurement invariance (MI) across time, which indicates that the same construct is measured at different time points [109]. On the other hand, different constructs may be unintentionally assessed at different time points, leading to biased results and subsequent errors in diagnosis and treatment selection.

Recently, Agtarap et al. [110] explored the RPQ’s MI longitudinally in the USA using a mild TBI sample. The authors found a general model comprising 16 items and 3 factors: emotional (irritable, depressed or frustrated), cognitive (forgetfulness, concentration or a longer time to think) and vison (blurred vision, light sensitive or double vision). This four-factor model provided excellent fit to their data and explicitly challenged the often-applied unidimensional structure of the RPQ. Nevertheless, no European studies on the longitudinal assessment of RPQ symptoms has been carried out so far. Since health care systems differ in Europe and the USA (i.e., most European countries have a free social security system, whereas the USA does not), the results of TBI studies conducted in the USA cannot be generalized to Europe.

To uncover the predictors, mechanisms and sequelae of TBI, a multi-site longitudinal cohort study called Collaborative European NeuroTrauma Effectiveness Research in TBI (CENTER-TBI; clinicaltrials.gov NCT0221022) collected data from patients after TBI in Europe and Israel. Among others, self-reported TBI symptoms were assessed at different time points following the TBI using the RPQ. 

Given the lack of empirical evidence on the longitudinal applications of the RPQ and the influence of sociodemographic, premorbid and injury-related factors on the (post-concussion) symptoms across time, in the present study, we aim to:

1. Analyze the longitudinal measurement invariance of RPQ symptoms from three to twelve months after TBI to verify that the RPQ measures the same construct at different time points following TBI.
2. Explore associations among sociodemographic, premorbid and injury-related factors and RPQ symptoms across time to model symptom trajectories for different subgroups of TBI patients.

## 2. Materials and Methods

### 2.1. Participants and Recruiting Procedure

The data were collected within the CENTER-TBI study at 63 centers across 18 countries in Europe and Israel from 19 December 2014 to 17 December 2017. Participants were included in the study whenever they received a clinical diagnosis of TBI, presented themselves within 24 h after injury and had for a positive computed tomography (CT) scan. Individuals were differentiated into three strata based on the admission type: emergency room (ER; evaluation at an ER only), admission (ADM; admission to a hospital ward), and intensive care unit (ICU; admission to an ICU). Ethical approval was obtained for each participating site (https://www.center-tbi.eu/project/ethical-approval, accessed on 12 July 2021), and informed consent was obtained from all participants or their legally authorized representatives. Further details can be found elsewhere [111].

**Figure 1:**
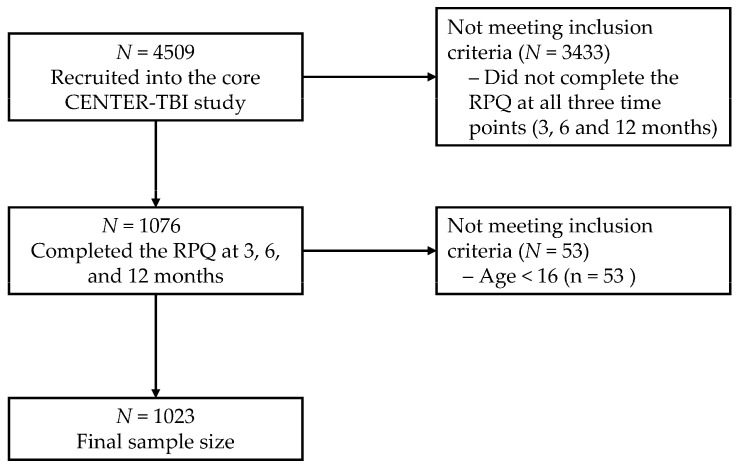
Sample flow chart diagram.

Data were retrieved from the CENTER-TBI database using the data access tool NEUROBOT, and the 3.0 core sample comprised 4509 participants. For the present study, we focused on individuals who had completed the RPQ assessments at three, six and twelve months after TBI. Due to the study design, individuals seen in the ER and then discharged were not involved in the twelve-month assessments. The present study included participants aged 16 and above, limited to those who filled out the RPQ at all three time points, yielding a final sample of 1023 participants. For more details, see Figure 1.

### 2.2. Measures

Sociodemographic and premorbid health history was collected upon enrollment in the study. Participants provided sociodemographic characteristics, such as sex, age, education level, marital and employment status as well as premorbid health history, such as previous concussions or TBIs and psychiatric problems prior to the TBI. 

Injury-related factors covered TBI and trauma severity. The severity of TBI was rated on the Glasgow Coma Scale (GCS) [112] in combination with the presence of CT abnormalities on the first CT scan (*uncomplicated mild*, GCS ≥ 13 and no CT abnormalities; *complicated mild*, GCS ≥ 13 and CT abnormalities present; *moderate*, GCS = 9–12; and *severe* TBI, GCS ≤ 8). The GCS was determined within the first 24 h post-injury. With the Injury Severity Score (ISS), trauma severity and polytrauma were evaluated by calculating the sum of the squares of the highest values of the three body regions measured by the Abbreviated Injury Scale score (AIS) [113].

The RPQ was used to assess 16 self-reported post-concussion symptoms (headaches, dizziness, nausea and/or vomiting, noise sensitivity, sleep disturbance, fatigue, irritability, depression, frustration, forgetfulness and poor memory, poor concentration, slow thinking, blurred vision, light sensitivity, double vision and restlessness). The RPQ total score ranges from 0 to 64 with cut-offs of 13, 25 and 33, indicating mild, moderate and severe symptoms, respectively [108]. 

### 2.3. Statistical Analyses

#### 2.3.1. Descriptive Analyses

The mean (*M*), median (*Mdn*), standard deviation (*SD*), skewness (*SK*; values |>3.0| indicate a severely skewed distribution) and kurtosis (*K*; values |>10.0| suggest significant deviation from a normal distribution [114]) were calculated for each RPQ symptom at three time points. The proportions of participants that reported “not experienced at all”, “no more of a problem (than before)”, “a mild problem”, “a moderate problem” and “a severe problem” were obtained for each symptom and time point. 

#### 2.3.2. RPQ Longitudinal Measurement Invariance

A longitudinal measurement invariance (MI) approach was chosen to assess differences in reported RPQ symptoms across time points. Based on the previous findings [107], we used the three-factor model comprising somatic, emotional and cognitive domains. To assess MI across three, six and twelve months, longitudinal confirmatory factor analysis (CFA) models were run for ordered categorical data [109]. For this purpose, three models with consecutive restrictions were estimated: (1) a baseline model/configural model, (2) a loading invariance model and (3) a threshold invariance model.

First, the configural model assumes that the same general pattern of factor loadings holds across time and assumes the following constraints:

- Latent intercepts are fixed to zero.
- The common factor mean is constrained to zero, and the unique factor covariance matrix is constrained to be 1.00.
- The same observed measure is chosen as the marker variable, and the factor loading of the marker variable is constrained to be 1.00.
- A threshold for each indicator is constrained to be equal across time.

Second, the loading invariance model adds to the configural model the constraint that factor loadings are identical across time. Third and finally, the threshold invariance model incorporates the constraint for each indicator, assuming that the threshold level of going from one response category to the next is identical across time. Loading and threshold invariance models were compared to baseline models using the following fit indices: the Comparative Fit Index (CFI) and the Tucker Lewis Index (TLI), where values above 0.95 establish adequate fit; the root-mean-square error of approximation (RMSEA; with a 90% confidence interval; CI_90%_) and the standardized root-mean-square residual (SRMR), where values < 0.06 to 0.08 establish adequate model fit [115]; and chi-squared difference statistics $\Delta X^{2}$. The type of estimator that was used was the diagonally weighted least square (DWLS) as an estimator commonly used with latent variable models with ordered categorical variables. This procedure was applied to each RPQ factor (somatic, emotional and cognitive), respectively.

#### 2.3.3. Demographic and Injury Characteristics Effects

To investigate the effects of demographic and injury characteristics, we estimated linear mixed models (LMMs) for each factor (somatic, emotional and cognitive) score and the conventional total RPQ score. We considered the following set of predictors:

1. Time since TBI (three, six and twelve months);
2. Sociodemographic factors: sex (female vs. male), age in years, education in years and interaction between sex and age;
3. Injury-related factors: TBI severity (uncomplicated mild TBI vs. more severe TBIs), injury cause (road traffic accident vs. fall vs. violent/other), admission type (ADM vs. ICU) and ISS [converted into log ISS distribution];
4. Premorbid factors: previous concussions (yes vs. no) and prior psychiatric problems (yes vs. no).

Three RPQ scale scores and the RPQ total score were used as dependent variables. Additionally, quadratic and cubic functions were evaluated for the time since TBI variable as well as for injury severity as measured by the ISS, and participants were included as random effects. For all significant predictors in LMMs, Type I error probability was set to 0.05 (one-tailed for testing directed hypotheses). To estimate the parameter values for LMMs, bootstrap estimation was used. All analyses were performed using R 4.0.5 [116]. The lme4 package [117] was used for LMMs, and the lavaan package [118] was used for longitudinal CFA. 

## 3. Results

Most participants were male (67.4%), with a mean age of 49.6 years (*SD* = 19.1; *Mdn* = 52.0; range 16 to 95). On average, they had 14.0 (*SD* = 4.1) years of education, and the majority were partnered (54.8%). Regarding injury characteristics, 45.0% of participants sustained complicated mild TBI, 50.8% were admitted to the ICU and the mean ISS was 22.1 (*SD* = 14.4). The demographic and injury characteristics of the participants are shown in Table 1.

Excluded participants (i.e., individuals younger than 16 years of age who did not complete the RPQ at all time points) did not differ systematically from participants included in analyses regarding sex (*X*^2^[1] = 2.79; *p* = 0.094), education level (*X*^2^[2] = 0.55; *p* = 0.757), age (*t* [2228] = −1.45; *p* = 0.145) or previous concussions (*X*^2^[1] = 0.17, *p* = 0.675). However, they differed with respect to extracranial injury severity level, according to ISS, (*X*^2^[3] = 32.36, *p* < 0.001), injury cause (*X*^2^[2] = 17.08, *p* < 0.001) and prior psychiatric problems (*X*^2^[1] = 6.26, *p* = 0.012). The excluded individuals more often had sustained mild injuries, were injured in road traffic accidents and had prior psychiatric problems. 

### 3.1. Descriptive Analyses

Regarding descriptive information on individual symptoms assessed three months after TBI, arithmetic means across participants ranged from 0.27 (nausea) to 1.65 (fatigue) with asymmetry (SK) of 0.13 (fatigue) to 2.97 (nausea and double vision). The same pattern was found at six and twelve months. Nausea had the lowest average values (0.20 and 0.23 at six and twelve months, respectively), and fatigue the highest (1.49 and 1.42 at six and twelve months, respectively) (see Appendix A, Table A1). 

An analysis of the proportions of each response category that was utilized indicated that the “not experienced at all” option was reported most often across all time points. The symptoms with the highest proportion in the category “mild problem” were fatigue (27% at three months and 24% at both six and twelve months), forgetfulness (21% at three months, 25% at six months and 22% at twelve months), poor concentration (21% at all three time points) and taking longer to think (20% at three months, 21% at six months and 20% at twelve months). Fatigue was the symptom with the highest proportion (8% in three months and 7% in six and twelve months) in the “severe problem” level (see Table 2). 

### 3.2. Longitudinal RPQ Measurement Invariance

The results from the MI testing for the three RPQ factors are presented in Table 3. The baseline model fit was adequate for the somatic factor (CFI = 0.942, TLI = 0.933, RMSEA = 0.059, CI_90%_ [0.056, 0.063]), indicating that the somatic-factorial structure represented the data well across all time points. There were no significant differences (*p* = 0.385) between the loading (CFI = 0.944, TLI = 0.938, RMSEA = 0.057 [0.054, 0.060]) and baseline model fit; therefore, intercepts were invariant across time. However, significant differences were found between the loading and threshold model fit (*p* < 0.001), indicating that the number of participants who reported each severity level can change over time. For example, the number of individuals who reported not experiencing fatigue at three months was 290, but this number increased to 345 at six months and to 372 at twelve months post-injury. However, from the initial 273 people who reported mild fatigue levels at three months, the number decreased to 242 people at six and twelve months, respectively (see the proportions for each level of response by time point in Table 2). 

Regarding the emotional factor, CFI = 0.999, TLI = 0.998, and RMSEA 0.022, CI_90%_ [0.009, 0.033] parameters showed adequate baseline model fit. In addition, no significant differences (*p* = 0.057) were found between the loading (CFI = 0.998, TLI = 0.998, RMSEA = 0.025 [0.014, 0.034]) and baseline model fit. However, significant differences (*p* < 0.001) were identified between the loading and threshold model fit (CFI = 0.977, TLI = 0.981, RMSEA = 0.070, CI_90%_ [0.064, 0.077]). Despite the differences, the threshold model showed adequate fit, which was slightly worse compared to the loading model fit.

Finally, for the cognitive factor, the same pattern was observed as for the previous factors. The baseline model fit was adequate across time (CFI = 1.000, TLI = 1.000, RMSEA < 0.001, CI_90%_ [0.000, 0.018]), and there were no significant differences between the loading and the baseline model fit (*p* = 0.376). Here, again, significant differences were found between the loading and threshold model fit (*p* < 0.001). 

Table A2 in Appendix A shows the discrepancies in the predicted probabilities between the threshold and loading invariance in each model. For example, for the somatic factor, symptom fatigue had the largest discrepancies in the predicted probabilities between the retained loading invariance model and the rejected threshold invariance model at three months after TBI [“No more of a problem (than before)” and “A mild problem”] and the symptom sleep disturbance at six months (“A moderate problem” and “A severe problem”).

### 3.3. Demographic and Injury Predictors of Factor Scores across 3, 6 and 12 Months

**Figure 2:**
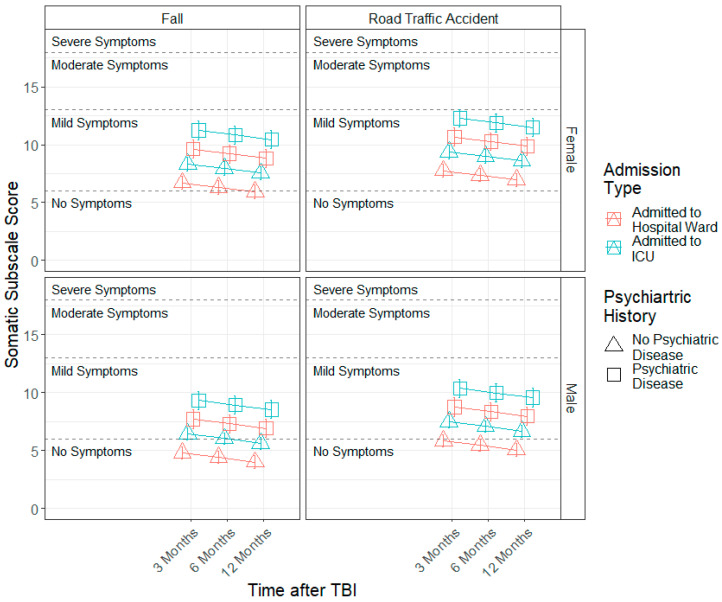
Modelled trajectories for the somatic factor by significant predictors.

**Figure 3:**
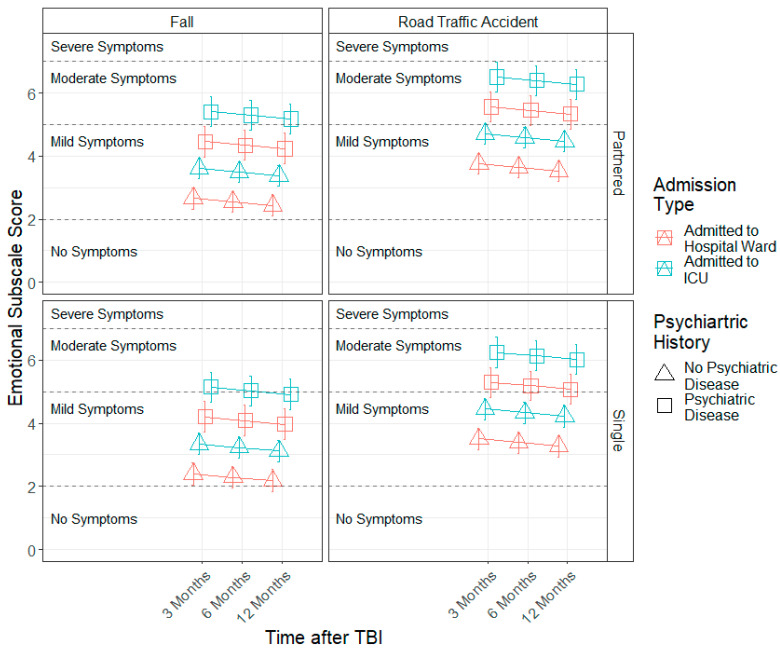
Modelled trajectories for the emotional factor by significant predictors.

The estimated models for each factor score can be found in Table 4. A significant effect of time since TBI (b = −0.79, SE = 0.17, *p* < 0.001) was found for the somatic factor, indicating that somatic symptom severity levels decreased linearly across time. Furthermore, sex (b = −2.55, SE = 1.10, *p* = 0.025), admission type (b = 1.62, SE = 0.55, *p* = 0.003), injury cause (b = −1.04, SE = 0.40, *p* = 0.014) and prior psychiatric problems (b = 2.90, SE = 0.61, *p* < 0.001) effects were found. Females, patients admitted to ICU, those who sustained a TBI by a road traffic accident or violence/other causes and individuals with prior psychiatric problems presented higher somatic symptom severity compared to males, patients admitted to hospital ward, those with falls as the injury cause and individuals who reported no prior psychiatric problems (see Figure 2). Similar results were found for the emotional factor (see Table 4 and Figure 3). 

**Figure 4:**
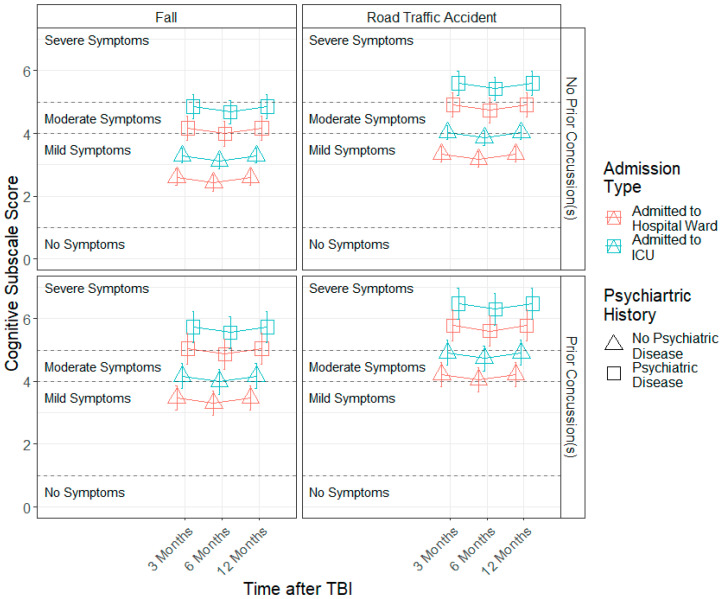
Modelled trajectories for the cognitive factor by significant predictors.

Regarding the cognitive factor, a significant quadratic time effect (b = 0.21, SE = 0.09, *p* = 0.037) was found, indicating that cognitive symptom severity levels decrease from 3 to 6 months, with an increase in severity from 6 to 12 months. Moreover, admission type (b = 0.69, SE = 0.31, *p* = 0.022), injury cause (b = −0.74, SE = 0.22, *p* = 0.001), prior concussions (b = 0.87, SE = 0.32, *p* = 0.010) and prior psychiatric problems (b = 1.56, SE = 0.34, *p* < 0.001) effects were found. Individuals who were admitted to the ICU, those who sustained a TBI as a result of a road accident and due to violence/other causes and patients with prior concussions and prior psychiatric problems presented higher cognitive symptom severity compared to patients admitted to a hospital ward, those with falls as the injury cause and patients without prior concussions and without prior psychiatric problems (see Figure 4).

**Figure 5:**
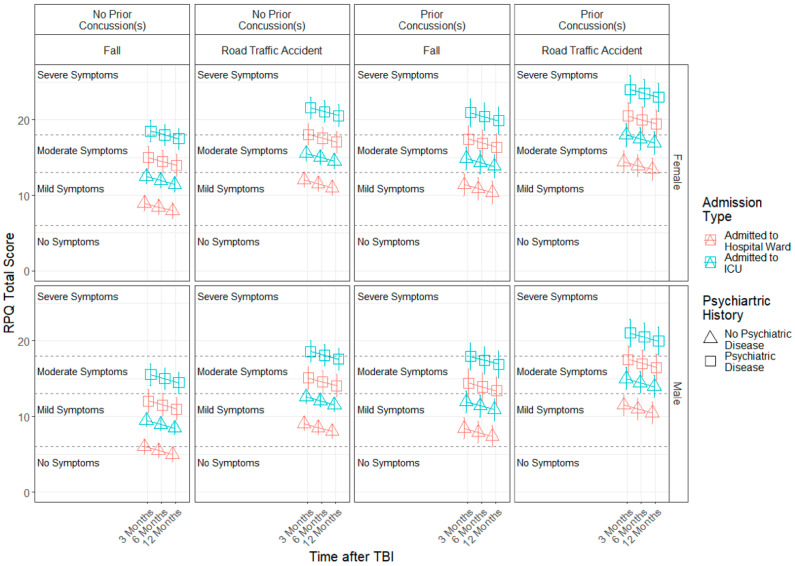
Modelled trajectories for the RPQ total score by significant predictors.

Finally, a significant effect of time since TBI (b = −1.03, SE = 0.31, *p* = 0.001) was determined with respect to the RPQ total score, showing that symptom severity levels decrease linearly from 3 to 12 months. Moreover, sex (b = −4.60, SE = 2.20, *p* = 0.038), admission type (b = 3.50, SE = 1.07, *p* = 0.001), injury cause (b = −3.07, SE = 0.85, *p* < 0.001), prior concussions (b = 2.42, SE = 1.23, *p* = 0.049) and psychiatric problems (b = 6.02, SE = 1.25, *p* < 0.001) effects were found. Females, patients who were admitted to the ICU, those who sustained a TBI caused by road traffic accidents or violence/other causes, patients with prior concussions and patients with prior psychiatric problems presented higher RPQ symptom severity compared to males, patients admitted to a hospital ward, those with falls as the injury cause, patients without a prior concussion and patients without prior psychiatric problems (see Figure 5).

## 4. Discussion

Given the lack of empirical evidence regarding the administration of the RPQ across time, the present study aimed to analyze patient-reported post-concussion symptoms longitudinally (three, six and twelve months). We investigated the measurement invariance assumption for the RPQ and associations between sociodemographic, premorbid and injury-related factors and RPQ symptoms within the first years after TBI using data obtained from the CENTER-TBI study. 

The results showed that the basic structure of the three factors remained stable across time (i.e., was invariant). In addition, factor loading changed longitudinally, and the proportion of symptoms reduced across time, with fatigue, poor concentration and taking longer to think being the most prevalent symptoms. Furthermore, we found that sex, injury cause and prior psychiatric problems were related to the somatic, emotional and cognitive domains as well as to the RPQ total score. 

Multiple scales have been used to assess post-TBI symptoms in research and clinical practice, with the RPQ being commonly applied in patients after TBI [62]. Despite its widespread use, very few studies have examined the RPQ longitudinally and using MI. Agtarap et al. [110] explored RPQ’s MI longitudinally in a mild TBI sample and found that a four-factor model provided the best model fit to their data. In our study, however, we employed the structural-factorial model defined by Smith-Seemiller et al. [102] based on theoretical considerations and the confirmation of its factorial structure in other studies using TBI sample (i.e., [62,104,106,108]). According to the MI philosophy, the structure of latent factors, which in this case is post-concussion symptomatology, should be stable or invariant, and the association between items and latent factors should not depend on group membership (e.g., a certain patient’s characteristics) or time [119]. Our results showed that post-concussion symptoms are clearly clustered in somatic, emotional and cognitive domains. Moreover, this structure was stable across the first year after TBI, regardless of its severity. Thus, even though a patient’s symptomatology changes across time, with increases or decreases in the number of symptoms and/or their intensity, clinicians and researchers can be sure that the RPQ retains its capacity to capture somatic, emotional and cognitive symptoms.

Regarding long-term symptom trajectories, the number and intensity of these symptoms tended to decline across time, although the few patients who reported severe problems concerning some symptoms at three months maintained the intensity of the problems at six and twelve months. These remaining long-term symptoms (e.g., fatigue and poor concentration) may be due to the difficulty that clinicians have with identifying and treating them, as there is a lack of scientifically proven standard protocols or strategies, [120] and it depends on the interaction of multiple factors (e.g., TBI severity, range of sequelae, patients’ coping strategies, etc.). Nevertheless, despite these change patterns in symptomatology (e.g., fatigue, sleep disturbance, forgetfulness, poor concentration and taking longer to think), the three-factor model fit was similar across time; therefore, RPQ can be considered a valid instrument to measure post-TBI symptomatology in individuals after TBI, with a broad severity range. 

Poor concentration and taking longer to think were the most prevalent symptoms among mild TBI patients across time, and fatigue was presented as the most prevalent in individuals with all severities (mild, moderate, and severe) for all time points. These results are consistent with several studies that have shown fatigue [3,11,15,24,45], poor concentration [11,15,24,29] and lower processing speed [3,11] as TBI residual symptoms. It is a shortcoming of the present study that ER patients (whose majority had mTBI) were excluded from the analysis due to the lack of complete data across time based on the study design (i.e., no assessments at 12 months after TBI). Including ER patients would have helped to interpret the relation between specific symptoms and TBI severity, as patients in ER strata were often found to report fewer post-concussion symptoms with lower intensity compared to those who were admitted to the hospital ward or the ICU [24].

Females, patients who were admitted to the ICU, those who sustained a TBI caused by a road traffic accident or violence/other causes, patients with prior concussions and patients with prior psychiatric problems presented higher RPQ symptom severity compared to males, patients admitted to the hospital ward, those with falls as the injury cause, patients without prior concussions and patients without prior psychiatric problems. It is, however, hard to judge the findings on factors associated with somatic, emotional and cognitive symptoms over the course of one year because of the cross-sectional nature and variety of applied measures in previous work. Most studies found that age [24,45,80,121,122], sex [24,45,81,121,122], education [11,24,45,122,123], employment status [24,80,122], TBI severity [24] and premorbid problems [15,24,80,123] are associated with RPQ symptomology, and thus, the present study would provide an extension of findings regarding these predictors over the course of one year. 

In the only longitudinal study, besides the present one, the authors found that time, sex, preinjury psychiatric disorders and race were related with RPQ measured symptomatology [110]. These results are partially consistent with our results because there was a linear decrease in the mean scores in the somatic and emotional symptoms and in the total score. However, the mean scores of the cognitive symptoms (forgetfulness, poor concentration and taking longer to think) increased slightly from 6 to 12 months, unlike the results found by Agtarap et al. [110]. This increase in cognitive symptoms may be because patients who incorporate activities into their daily life one year after injury realize their cognitive limitations and the challenges that they must face. Apart from the differing results concerning cognitive symptoms, we found that female patients and patients with prior psychiatric disorders reported higher symptom scores, which is in line with the findings of Agtarap et al. [110]. To further validate our results from the current study, the data from the TRACK-TBI study could be used. Such analyses, wherein we could also control for geographic regions, would shed more light on the longitudinal prevalence of symptoms after TBI and on potential protective and risk factors.

### 4.1. Limitations

We included as associated variables age, sex, etc., which are non-modifiable factors. Future studies should include modifiable predictors (e.g., the type of rehabilitation received, the number of rehabilitation hours received, and social support). Even though all patients were from Europe or Israel, the health care system varies across these countries and may impact these outcomes, so future studies should consider including country as a predictor, if the numbers of participants are high enough. Furthermore, the distribution of regions was unequal, with Eastern Europe being the most underrepresented and thus contributing minimally to the study results. Additionally, as individuals admitted to the ER were discharged before the twelve-month assessments, they were excluded from the analyses. Future studies should include information of this type of patient to verify the invariant structure of the latent factors of RPQ. Finally, we did not have information on whether patients had suffered a further TBI during the period of follow-up, which may have influenced the number of symptoms reported.

### 4.2. Implications

First, our results indicate that the questionnaire retains its factorial structure over time and thus can be implemented into longitudinal evaluations, follow up assessments and diagnostic protocols to identify and associate RPQ symptoms during the first year post-injury in a variety of individuals with different ranges of TBI severity. Second, clinicians should pay attention to potential at-risk groups. Females, patients admitted to the ICU, those who sustained a TBI due to a road traffic accident and violence/other causes and individuals with prior psychiatric problems appear to be more likely to report persistent symptoms (post-concussion) and should be diagnosed and treated appropriately in a timely manner. Overall, the early identification of these symptoms and their associated variables may help to implement early, customizable interventions to prevent future complications, sequalae or the chronification of symptoms.

The results of our study provide evidence that the RPQ retains its factorial structure over a period of one year. This conclusion is supported by a good fitting three-factor model in both cross-sectional [107] and longitudinal studies; therefore, researchers and clinicians can use robust symptom factors in their clinical work or research studies.

## 5. Conclusions

The aim of this study was to analyze the RPQ’s measurement invariance within the first year after TBI and associated variables related to RPQ symptoms. The results showed that the three-factor structure comprising somatic, emotional and cognitive domains remains stable across time and that the proportion of symptoms reduced during the first year after injury, but fatigue, poor concentration and taking longer to think persisted as the most prevalent symptoms. Therefore, it is of utmost importance to ensure the adequate diagnosis and treatment of persistent symptoms to facilitate the return to daily life for those affected. Moreover, certain subgroups of patients who are at higher risk of experiencing symptoms over time should be treated in a timely and appropriate manner. Thus, researchers and clinicians now have evidence that the RPQ retains its factorial structure over a period of one year, allowing the identification of symptoms and their severity range during that period. Furthermore, the identification of associated factors may help to prevent future complications of the symptomatology after TBI. 

## Figures and Tables

**Table 1:** Demographic characteristics of the sample.

Variable	Level	*n*	%	Mean (SD)
Age in years	--	--	--	49.6 (19.1)
Education in years	--	--	--	14.0 (4.1)
Sex	Female	334	(32.6%)	--
	Male	689	(67.4%)	--
Race	Asian	11	(1%)	--
	Black	8	(1%)	--
	White	982	(96%)	--
	Missing	23	(2%)	--
Marital status	Partnered	560	(54.8%)	--
	Single	461	(45.8%)	--
Employment status	Full-time employed	447	(46.5%)	--
	Part-time employed	111	(11.5%)	--
	In training	100	(10.4%)	--
	Unemployed	67	(7.0%)	--
	Retired	237	(24.6%)	--
Geographical region	Eastern Europe	8	(1%)	--
	Northern Europe	299	(29%)	--
	Southern Europe	265	(26%)	--
	Western Europe	451	(44%)	--
TBI severity	Uncomplicated	233	(24.6%)	--
	Mild	427	(45.0%)	--
	Moderate	93	(9.8%)	--
	Severe	196	(20.7%)	--
Injury cause	Road traffic accident	454	(45.4%)	--
	Fall	410	(41.0%)	--
	Violent/other	137	(13.7%)	--
Admission type	ADM	503	(49.2%)	--
	ICU	520	(50.8%)	--
Previous concussions	No	884	(90.2%)	--
	Yes	96	(9.8%)	--
Prior psychiatric problems	No	909	(89.6%)	--
	Yes	106	(10.4%)	--

Note: ADM = Admission to a hospital ward; ICU = Intensive care unit; Geographical region = Western Europe (Austria, Belgium, France, Germany, the Netherlands, and United Kingdom); Northern Europe (Denmark, Finland, Latvia, Lithuania, Norway and Sweden); Southern/Eastern Europe (Italy, Spain, Hungary, Romania, and Serbia); Race = Asian (Asian Far East, Asian South and Asian other); Black (Black African and Black Afro-Caribbean), White = (White, White African, White Australian, White European, White Middle-Eastern, White North American, White South American, and White other).

**Table 2:** Proportions for each level of response by time point (3, 6 and 12 months).

	N = 1023		Not Experienced at All			No More of a Problem (Than Before)			A Mild Problem			A Moderate Problem			A Severe Problem		
Item	Abbreviation ^1^	Scale	3M	6M	12M	3M	6M	12M	3M	6M	12M	3M	6M	12M	3M	6M	12M
Headaches	Headaches	S	0.5	0.55	0.57	0.18	0.18	0.17	0.18	0.15	0.15	0.12	0.11	0.09	0.02	0.02	0.03
Feeling of Dizziness	Dizziness	S	0.54	0.59	0.6	0.13	0.14	0.16	0.19	0.17	0.15	0.09	0.08	0.07	0.04	0.03	0.03
Nausea and/or Vomiting	Nausea	S	0.84	0.88	0.85	0.08	0.07	0.09	0.05	0.04	0.04	0.02	0.02	0.01	0.01	0	0.01
Noise Sensitivity, easily upset by loud noise	Noise Sensitivity	S	0.6	0.61	0.61	0.14	0.14	0.13	0.14	0.14	0.13	0.09	0.08	0.09	0.03	0.03	0.04
Sleep Disturbance	Sleep Disturbance	S	0.5	0.53	0.51	0.19	0.18	0.2	0.15	0.15	0.14	0.1	0.1	0.1	0.06	0.04	0.06
Fatigue, tiring more easily	Fatigue	S	0.28	0.34	0.36	0.16	0.17	0.16	0.27	0.24	0.24	0.21	0.18	0.17	0.08	0.07	0.07
Being Irritable, easily angered	Irritable	E	0.49	0.51	0.49	0.23	0.21	0.23	0.15	0.17	0.17	0.1	0.09	0.09	0.03	0.03	0.02
Feeling Depressed or Tearful	Depressed	E	0.54	0.55	0.55	0.17	0.19	0.18	0.15	0.14	0.15	0.11	0.09	0.09	0.02	0.02	0.03
Feeling Frustrated or Impatient	Frustrated	E	0.46	0.5	0.51	0.21	0.22	0.22	0.17	0.16	0.15	0.13	0.1	0.09	0.04	0.03	0.03
Forgetfulness, poor memory	Forgetful	C	0.37	0.38	0.37	0.23	0.21	0.22	0.21	0.25	0.22	0.13	0.12	0.13	0.05	0.04	0.06
Poor Concentration	Poor Concentration	C	0.42	0.44	0.42	0.2	0.2	0.21	0.21	0.21	0.21	0.13	0.12	0.12	0.04	0.03	0.04
Taking Longer to Think	Longer to Think	C	0.47	0.48	0.47	0.18	0.18	0.19	0.2	0.21	0.2	0.11	0.1	0.11	0.04	0.03	0.04
Blurred Vision	Blurred Vision	S	0.67	0.69	0.72	0.15	0.14	0.14	0.09	0.09	0.09	0.06	0.05	0.03	0.03	0.03	0.02
Light Sensitivity, easily upset by bright light	Light Sensitivity	S	0.7	0.71	0.68	0.14	0.13	0.16	0.1	0.09	0.1	0.04	0.05	0.05	0.02	0.02	0.02
Double Vision	Double Vision	S	0.83	0.84	0.84	0.07	0.07	0.09	0.04	0.05	0.04	0.03	0.02	0.02	0.03	0.02	0.01
Restlessness	Restless	E	0.61	0.63	0.61	0.17	0.18	0.18	0.14	0.11	0.14	0.07	0.06	0.05	0.01	0.02	0.02

^1^ For simplicity, abbreviations for items appear throughout the manuscript. Note: S = somatic factor, E = emotional factor, C = cognitive factor.

**Table 3:** Results from the longitudinal measurement invariance tests for the factors using the DWLS.

Factor	Model	Robust Goodness-of-Fit			CFI	TLI	RMSEA [CI_90%_]	SRMR	$X^{2}$		
		$X^{2}$	*df*	*p*					$\Delta X^{2}$	Δdf	Δp
Somatic	Baseline	1395.998	303	<0.001	0.942	0.933	0.059 [0.056, 0.063]	0.069	--	--	--
	Loading	1388.77	319	<0.001	0.944	0.938	0.057 [0.054, 0.060]	0.069	17	16	0.385
	Threshold	1656.264	362	<0.001	0.932	0.934	0.059 [0.056, 0.062]	0.07	2390.9	43	<0.001
Emotional	Baseline	63.169	42	0.964	0.999	0.998	0.022 [0.009, 0.033]	0.015	--	--	--
	Loading	77.636	48	0.004	0.998	0.998	0.025 [0.014, 0.034]	0.015	120.22	6	0.057
	Threshold	479.446	79	<0.001	0.977	0.981	0.070 [0.064, 0.077]	0.044	3540.8	31	<0.001
Cognitive	Baseline	12.914	18	0.797	10	10	0.000 [0.000, 0.018]	0.006	--	--	--
	Loading	17.947	22	0.709	10	10	0.000 [0.000, 0.020]	0.006	40.226	4	0.376
	Threshold	287.322	44	<0.001	0.99	0.992	0.074 [0.066, 0.082]	0.033	2390.6	22	<0.001

Note: $\left(\Delta \right)X^{2}$ = chi-squared value (of the difference test); (Δ)*df* = degrees of freedom (of the difference test); (Δ)*p* = *p*-value (of the difference test); CFI = comparative fit index; TLI = Tucker-Lewis Index; RMSEA = root-mean-square error of approximation, including the 90% confidence interval (CI); SRMR = standardized root-mean-square residual.

**Table 4:** Demographic and injury predictors of factor score trajectories across 3, 6 and 12 months.

	Somatic	Emotional	Cognitive	Total
Fixed Effects (Reference Group)	*β* (SE) ^†^	*Β* (SE) ^†^	*Β* (SE) ^†^	*Β* (SE) ^†^
Intercept	7.76 (1.67) ***	3.96 (1.03) ***	3.47 (0.89) ***	13.24 (3.27) ***
Time point Linear	−0.79 (0.17) ***	−0.22 (0.11) *	−0.006 (0.09)	−1.03 (0.31) **
Time point Quadratic	0.26 (0.20)	0.20 (0.11)	0.21 (0.09) *	0.52 (0.33)
Age in years	0.002 (0.01)	−0.006 (0.01)	0.004 (0.01)	−0.01 (0.04)
Education in years	−0.04 (0.04)	−0.04 (0.03)	−0.02 (0.02)	−0.09 (0.09)
Sex (Male)	−2.55 (1.10) *	−1.57 (0.72)	−0.77 (0.58)	−4.60 (2.20) *
Sex: Age	0.01 (0.02)	0.01 (0.01)	0.005 (0.01)	0.03 (0.04)
Marital State (Single)	0.003 (0.42)	−0.26 (0.25) *	0.08 (0.22)	−0.39 (0.84)
Severity (GCS + CT)—Linear	−0.65 (0.81)	−0.05 (0.48)	0.74 (0.46)	0.67 (1.57)
Severity (GCS + CT)—Quadratic	−0.02 (0.85)	−0.25 (0.52)	0.53 (0.49)	0.08 (1.99)
Severity (GCS + CT)—Cubic	−0.006 (0.76)	0.16 (0.43)	0.39 (0.45)	0.80 (1.61)
Injury cause (Fall)	−1.04 (0.40) *	−1.10 (0.25) ***	−0.74 (0.22) **	−3.07 (0.85) ***
Injury cause (Violent/other)	−0.01 (0.64)	−0.04 (0.38)	−0.43 (0.32)	−0.64 (1.17)
Admission type (ICU)	1.62 (0.55) **	0.94 (0.33) **	0.69 (0.31) *	3.50 (1.07) **
Total ISS	−0.03 (0.34)	0.27 (0.25)	0.03 (0.19)	0.15 (0.73)
Previous concussions (yes)	0.50 (0.62)	0.39 (0.39)	0.87 (0.32) *	2.42 (1.23) *
Prior psychiatric problems (yes)	2.90 (0.61) ***	1.80(0.38) ***	1.56 (0.34) ***	6.02 (1.25) ***

Note: β = Regression coefficients; SE = Standard errors; ^†^ = β and SE values for LMMs bootstrap estimation was used; ICU = Intensive care unit; Sex: Age = interaction term; *** *p* < 0.001; ** *p* < 0.005; * *p* < 0.05; Severity was rated using the Glasgow Coma Scale (GCS) and computed tomography (CT).

## Data Availability

All relevant data are available upon request from CENTER-TBI, and the authors are not legally allowed to share it publicly. The authors confirm that they received no special access privileges to the data. CENTER-TBI is committed to data sharing and, in particular, to responsible further use of the data. Hereto, we have a data sharing statement in place: https://www.center-tbi.eu/data/sharing (accessed on 11 July 2022). The CENTER-TBI Management Committee, in collaboration with the General Assembly, established the Data Sharing Policy and the Publication and Authorship Guidelines to assure the correct and appropriate use of the data, as the dataset is hugely complex and requires the help of experts from the Data Curation Team or Bio- Statistical Team for correct use. This means that we encourage researchers to contact the CENTER-TBI team for any research plans and the Data Curation Team for any help in the appropriate use of the data, including the sharing of scripts. Requests for data access can be submitted online: https://www.center-tbi.eu/data (accessed on 11 July 2022). The complete manual for data access is also available online: https://www.center-tbi.eu/files/SOP-Manual-DAPR-2402020.pdf (accessed on 11 July 2022).

## Supplementary Materials

The following supporting information can be downloaded at: www.mdpi.com/article/10.3390/jcm11144090/s1. The full list of the CENTER-TBI participants and investigators can be found in the Online Supplement.

## Appendix A

**Table A1:** Descriptive data analyses by item and time point.

Time Point	Item	Mean	SD	Median	Skew	Kurtosis	SE
3M	Headaches	0.99	1.17	0	0.82	−0.58	0.04
	Dizziness	0.96	1.22	0	0.95	−0.31	0.04
	Nausea	0.27	0.7	0	2.97	9.01	0.02
	Noise Sensitivity	0.8	1.14	0	1.19	0.21	0.04
	Sleep Disturbance	1.02	1.25	0	0.98	−0.25	0.04
	Fatigue	1.65	1.31	2	0.13	−1.17	0.04
	Irritable	0.94	1.13	1	0.98	−0.10	0.04
	Depressed	0.91	1.16	0	0.98	−0.28	0.04
	Frustrated	1.06	1.2	1	0.81	−0.55	0.04
	Forgetful	1.27	1.24	1	0.58	−0.79	0.04
	Poor Concentration	1.16	1.21	1	0.65	−0.74	0.04
	Longer to Think	1.05	1.2	1	0.78	−0.57	0.04
	Blurred Vision	0.63	1.07	0	1.66	1.75	0.03
	Light Sensitivity	0.53	0.95	0	1.82	2.6	0.03
	Double Vision	0.35	0.9	0	2.79	7.07	0.03
	Restless	0.71	1.03	0	1.29	0.64	0.03
6M	Headaches	0.88	1.14	0	1	−0.22	0.04
	Dizziness	0.82	1.13	0	1.13	0.15	0.04
	Nausea	0.2	0.59	0	3.22	10.07	0.02
	Noise Sensitivity	0.78	1.14	0	1.28	0.5	0.04
	Sleep Disturbance	0.93	1.19	0	1.03	−0.11	0.04
	Fatigue	1.49	1.32	1	0.31	−1.15	0.04
	Irritable	0.93	1.13	0	0.97	−0.13	0.04
	Depressed	0.85	1.13	0	1.09	0.06	0.04
	Frustrated	0.95	1.14	1	0.95	−0.20	0.04
	Forgetful	1.24	1.19	1	0.53	−0.79	0.04
	Poor Concentration	1.09	1.17	1	0.69	−0.69	0.04
	Longer to Think	1.02	1.16	1	0.78	−0.53	0.04
	Blurred Vision	0.6	1.05	0	1.77	2.2	0.03
	Light Sensitivity	0.54	0.98	0	1.84	2.54	0.03
	Double Vision	0.32	0.84	0	2.95	8.37	0.03
	Restless	0.65	1	0	1.5	1.39	0.03
12M	Headaches	0.84	1.14	0	1.11	0.07	0.04
	Dizziness	0.76	1.09	0	1.26	0.55	0.03
	Nausea	0.23	0.63	0	3.3	12.02	0.02
	Noise Sensitivity	0.83	1.21	0	1.21	0.22	0.04
	Sleep Disturbance	1.01	1.25	0	0.99	−0.23	0.04
	Fatigue	1.42	1.32	1	0.39	−1.10	0.04
	Irritable	0.93	1.1	1	0.94	−0.14	0.03
	Depressed	0.87	1.15	0	1.1	0.07	0.04
	Frustrated	0.93	1.14	0	1.01	−0.05	0.04
	Forgetful	1.28	1.25	1	0.58	−0.79	0.04
	Poor Concentration	1.17	1.22	1	0.67	−0.67	0.04
	Longer to Think	1.06	1.2	1	0.8	−0.49	0.04
	Blurred Vision	0.49	0.93	0	2.04	3.64	0.03
	Light Sensitivity	0.56	0.96	0	1.72	2.16	0.03
	Double Vision	0.28	0.75	0	3.15	10.19	0.02
	Restless	0.69	1.01	0	1.37	1.02	0.03

Note: SD = standard deviation; Skew = skewness; SE = standard error; 3M = three months; 6M = six months; 12M = twelve months.

**Table A2:** Discrepancies in predicted probabilities based on threshold invariance vs. loading invariance models for each factor.

Factor	Time Point	Indicator/Item	Not Experienced at All	No More of a Problem (Than Before)	A Mild Problem	A Moderate Problem	A Severe Problem
Somatic	3M	Headaches	0.02	−0.06	−0.02	0.03	0.02
		Dizziness	0	−0.04	−0.01	0.02	0.03
		Nausea	−0.06	0.02	0.02	0.01	0.01
		Noise Sensitivity	−0.07	−0.04	0.03	0.04	0.04
		Sleep Disturbance	**−0.11**	0.06	−0.01	0.03	0.03
		Fatigue	0.2	**−0.15**	**−0.15**	0.02	0.07
		Blurred Vision	−0.05	−0.02	0.02	0.02	0.03
		Light Sensitivity	**−0.12**	0.02	0.05	0.04	0.02
		Double Vision	−0.06	0.02	0.02	0.01	0.02
	6M	Headaches	0	0	0.01	0	0
		Dizziness	0	0	0	0	0
		Nausea	−0.01	0.01	0	0.01	−0.01
		Noise Sensitivity	0	0	0	−0.09	0.09
		Sleep Disturbance	0	−0.01	0	**0.12**	**−0.12**
		Fatigue	0	0.01	0	0.02	−0.03
		Blurred Vision	0	0.01	−0.01	0	0.01
		Light Sensitivity	0	0	0	−0.01	0.01
		Double Vision	0	−0.01	0.01	0	0
	12M	Headaches	−0.01	0.01	0	0	0
		Dizziness	0	0	0	0	0
		Nausea	0.01	−0.01	0	0	0
		Noise Sensitivity	0.02	0	−0.02	0.01	−0.01
		Sleep Disturbance	−0.06	0.06	−0.01	−0.01	0
		Fatigue	0.07	−0.09	0.02	0.01	−0.01
		Blurred Vision	−0.01	0.01	−0.01	0.01	0
		Light Sensitivity	0.03	−0.03	0	0	0
		Double Vision	0.01	−0.01	0	0	0
Emotional	3M	Irritable	−0.01	−0.09	0	0.07	0.03
		Depressed	−0.04	−0.09	0.03	0.08	0.03
		Frustrated	0.06	−0.23	0.04	0.1	0.03
		Restless	−0.07	−0.04	0.04	0.05	0.02
	6M	Irritable	0	**−0.11**	0.03	0.06	0.02
		Depressed	−0.03	−0.10	0.02	0.08	0.03
		Frustrated	0.01	**−0.20**	0.06	0.1	0.03
		Restless	−0.09	−0.04	0.07	0.05	0.01
	12M	Irritable	0.01	**−0.13**	0.02	0.07	0.02
		Depressed	−0.06	**−0.10**	0.06	0.08	0.02
		Frustrated	−0.04	**−0.19**	**0.11**	0.1	0.03
		Restless	−0.06	−0.05	0.05	0.05	0.01
Cognitive	3M	Forgetful	0	0	0	0	0
		Poor Concentration	0.01	−0.01	0	0	0
		Longer to Think	0	0	0	0	0
	6M	Forgetful	0.09	**−0.16**	−0.07	0.09	0.04
		Poor Concentration	0.09	**−0.28**	0.05	**0.11**	0.03
		Longer to Think	0.02	**−0.18**	0.03	0.09	0.03
	12M	Forgetful	0.03	−0.01	0	−0.01	0
		Poor Concentration	0.03	−0.02	−0.01	0	0
		Longer to Think	0.03	−0.02	−0.01	0	0

Note: **Bolded** values are those that represent discrepancies greater than 0.10 in absolute values. The calculation of these probabilities is based on the ordered categorical confirmatory factor analysis output from the *lavaan* package using diagonally weighted least squares with robust correction; 3M = three months; 6M = six months; 12M = twelve months.
